# RNA Editing During Sexual Development Occurs in Distantly Related Filamentous Ascomycetes

**DOI:** 10.1093/gbe/evx052

**Published:** 2017-04-01

**Authors:** Ines Teichert, Tim A. Dahlmann, Ulrich Kück, Minou Nowrousian

**Affiliations:** Lehrstuhl für Allgemeine und Molekulare Botanik, Ruhr-Universität Bochum, Germany

**Keywords:** A-to-I RNA editing, *Sordaria macrospora*, *Pyronema confluens*, fruiting body, protoperithecia, sexual development

## Abstract

RNA editing is a post-transcriptional process that modifies RNA molecules leading to transcript sequences that differ from their template DNA. A-to-I editing was found to be widely distributed in nuclear transcripts of metazoa, but was detected in fungi only recently in a study of the filamentous ascomycete *Fusarium graminearum* that revealed extensive A-to-I editing of mRNAs in sexual structures (fruiting bodies). Here, we searched for putative RNA editing events in RNA-seq data from *Sordaria macrospora* and *Pyronema confluens*, two distantly related filamentous ascomycetes, and in data from the Taphrinomycete *Schizosaccharomyces pombe*. Like *F. graminearum*, *S. macrospora* is a member of the Sordariomycetes, whereas *P. confluens* belongs to the early-diverging group of Pezizomycetes. We found extensive A-to-I editing in RNA-seq data from sexual mycelium from both filamentous ascomycetes, but not in vegetative structures. A-to-I editing was not detected in different stages of meiosis of *S. pombe*. A comparison of A-to-I editing in *S. macrospora* with *F. graminearum* and *P. confluens*, respectively, revealed little conservation of individual editing sites. An analysis of RNA-seq data from two sterile developmental mutants of *S. macrospora* showed that A-to-I editing is strongly reduced in these strains. Sequencing of cDNA fragments containing more than one editing site from *P. confluens* showed that at the beginning of sexual development, transcripts were incompletely edited or unedited, whereas in later stages transcripts were more extensively edited. Taken together, these data suggest that A-to-I RNA editing is an evolutionary conserved feature during fruiting body development in filamentous ascomycetes.

## Introduction

Most eukaryotic RNAs undergo modifications after transcription, for example splicing or capping. One such modification is RNA editing, which describes post-transcriptional changes in RNA molecules leading to transcript sequences that differ in sequence from their template DNA. The definition includes insertion, deletion, or modification of nucleotides, but excludes events like splicing, capping, polyadenylation etc. (Gott 2003, Farajollahi and Maas 2010, Knoop 2011). RNA editing was first discovered in the mitochondrial DNA of Trypanosomes, but has since been found to be present in a wide range of eukaryotic groups and in all DNA-containing organelles (Knoop 2011). However, editing of nuclear-encoded protein-coding genes has until recently been described only in metazoa (multicellular animals). Most prevalent in metazoan nuclei is A-to-I editing, in which an adenosine deaminase acting on RNA (ADAR) enzyme converts an adenosine to inosine (Bass 2002, Jin et al. 2009, Grice and Degnan 2015). During translation, inosine is interpreted as guanosine (Basilio et al. 1962), effectively changing the genetic code from A to G at edited sites within protein-coding regions. However, the effect of A-to-I editing on coding capacity varies greatly in different species. In humans, for example, only few A-to-I editing sites lead to codon changes (Bahn et al. 2012, Bazak et al. 2014), whereas in the squid *Doryteuthis pealeii*, the majority of investigated protein-coding genes undergo recoding through RNA editing (Alon et al. 2015).

In fungi, nuclear RNA editing was only recently investigated in the basidiomycete *Ganoderma lucidum* and in the filamentous ascomycete *Fusarium graminearum* (Zhu et al. 2014, Liu et al. 2016). In *G. lucidum*, about 8900 putative editing sites were identified based on RNA-seq data, but no preference for A-to-I over other forms of editing was found, and the functional significance of editing was not studied (Zhu et al. 2014). In *F. graminearum*, A-to-I RNA editing was shown to occur during sexual development, with more than 26,000 editing sites specific to fruiting bodies (perithecia). Furthermore, A-to-I RNA editing in this species is important for sexual development, as it was demonstrated that only an edited version of the protein kinase PUK1 is functional and allows wild type-like ascospore morphology and discharge (Liu et al. 2016). The authors also found genome-wide A-to-I editing in *Fusarium verticilloides*, and editing of the *puk1* ortholog in the related species *Neurospora crassa* (Liu et al. 2016). However, it is not clear how widespread RNA editing is in filamentous ascomycetes, and if there is a general A-to-I prevalence. Furthermore, it is not clear if RNA editing in other ascomycete groups is also tied to sexual development.

Here, we analyzed the potential for RNA editing in several developmental stages of two additional filamentous ascomycetes, *Sordaria macrospora* and *Pyronema confluens*. While *S. macrospora* is a Sordariomycete and develops perithecia as fruiting bodies similar to *F. graminearum*, *P. confluens* is a member of the Pezizomycetes, one of the earliest-diverging lineages of filamentous ascomycetes that produce apothecia as fruiting bodies (Gwynne-Vaughan and Williamson 1931, Teichert et al. 2014). *Fusarium graminearum*, *S. macrospora*, and *P. confluens* are all homothallic, that is self-fertile, and therefore able to undergo the sexual cycle without the need for a mating partner. We analyzed RNA-seq data from samples of mycelia from vegetative and sexual stages of *S. macrospora* and *P. confluens*. In addition, we analyzed data from two developmental mutants of *S. macrospora* that are blocked at an early stage of fruiting body formation. Our analyses show that A-to-I editing is prevalent during sexual development in both species, but mostly absent during vegetative growth as well as in the young fruiting bodies (protoperithecia) of the *S. macrospora* developmental mutants. These data suggest that A-to-I RNA editing is conserved during sexual development in filamentous ascomycetes.

## Materials and Methods

### Strains and Growth Conditions

The strains used in this study were the *S. macrospora* wild type (strain S133143 from our laboratory collection) and developmental mutant pro1 (Masloff et al. 1999), as well as the *P. confluens* wild type strain (CBS 100304). Unless stated otherwise, standard growth conditions for *S. macrospora* were as described (Masloff et al. 1999, Nowrousian et al. 1999). For RNA extraction from cultures undergoing sexual development, *S. macrospora* was grown at 25 °C in minimal medium in surface cultures as described (Nowrousian et al. 2005). *Pyronema confluens* was grown on minimal medium as previously described (Nowrousian and Kück 2006).

### Bioinformatics Analysis of RNA Editing in *S. macrospora*, *P. confluens*, and *Schizosaccharomyces pombe*

Publicly available RNA-seq data (table 1) were analyzed for the presence of putative editing sites. First, raw sequence reads from *S. macrospora* and *P. confluens* were trimmed to remove undetermined bases and polyA/polyT stretches from the ends, and quality trimming from the 3' and 5' end was performed until the base quality score was at least 10. Trimmed reads of at least 40 bases were mapped onto the predicted gene sequences (coding sequences and untranslated regions including introns) based on the genome annotation of *S. macrospora* and *P. confluens* (Nowrousian et al. 2010, Teichert et al. 2012, Traeger et al. 2013) using Tophat version 2.1.1 (Kim et al. 2013). The shorter reads of *S. pombe* (Wilhelm et al. 2008) were mapped directly without trimming. Reads were mapped onto gene sequences (including coding sequences, introns, and untranslated regions) instead of genome sequences to be able to directly identify A-to-G changes (when mapping onto genome sequences, A-to-G changes in genes encoded on the reverse strand would appear as T-to-C instead). Based on the mapped reads, the mpileup function of SAMtools (Li et al. 2009) was used to generate coverage information for each base in the predicted RNAs for each of the analyzed samples. Custom-made Perl scripts were used to identify putative sequence variants from the coverage information. Variants were filtered for putative editing sites using custom-made Perl scripts that retained only variants with a single alternative base (i.e. no insertions/deletions or positions with more than one base difference from the reference genome), a minimal coverage of five reads (*S. macrospora*, *P. confluens*) or three reads (*S. pombe*, the lower threshold was used due to lower read coverage), at least 3% and two reads coverage of the alternative base, similar to the conditions used in a previous study for *F. graminearum* (Liu et al. 2016). It was then analyzed which variants were present in both independent biological replicates available for each analyzed condition (table 1), and only these reproducibly identified variants were analyzed further using custom-made Perl scripts.

**Table 1:** GEO and Array Express Accession Numbers of RNA-Seq Data Used in This Study

Species	Strain	Condition^a^	Accession Numbers^b^	Reference
*Sordaria macrospora*	wild type	vegetative mycelium	GSE33668	(Teichert et al. 2012)
*Sordaria macrospora*	wild type	sexual mycelium	GSE33668	(Teichert et al. 2012)
*Sordaria macrospora*	wild type	protoperithecia	GSE33668	(Teichert et al. 2012)
*Sordaria macrospora*	pro1	protoperithecia	GSE33668	(Teichert et al. 2012)
*Sordaria macrospora*	nox1	protoperithecia	GSE49363	(Dirschnabel et al. 2014)
*Pyronema confluens*	wild type	sex	GSE41631	(Traeger et al. 2013)
*Pyronema confluens*	wild type	DD	GSE41631	(Traeger et al. 2013)
*Pyronema confluens*	wild type	vegmix	GSE41631	(Traeger et al. 2013)
*Schizosaccharomyces pombe*	JB22 972h-	vegetative growth	E-MTAB-5	(Wilhelm et al. 2008)
*Schizosaccharomyces pombe*	JB371	meiosis 0 h	E-MTAB-5	(Wilhelm et al. 2008)
*Schizosaccharomyces pombe*	JB371	meiosis 1 + 2 h	E-MTAB-5	(Wilhelm et al. 2008)
*Schizosaccharomyces pombe*	JB371	meiosis 3 + 4 h	E-MTAB-5	(Wilhelm et al. 2008)
*Schizosaccharomyces pombe*	JB371	meiosis 5 + 6 h	E-MTAB-5	(Wilhelm et al. 2008)
*Schizosaccharomyces pombe*	JB371	meiosis 7 + 8 h	E-MTAB-5	(Wilhelm et al. 2008)

a For each condition, RNA-seq data from two independent biological replicates are available. Growth conditions for *S. macrospora* represent total sexual or vegetative mycelium (grown as surface culture or submerged, respectively, in liquid medium) or young fruiting bodies (protoperithecia) that were isolated by laser microdissection (Teichert et al. 2012). Growth conditions for *P. confluens* represent sexual development (sex), long-term culturing in the dark which prevents sexual development (DD), and a mixture of different vegetative tissues from different growth conditions that prevent sexual development (vegmix) (Traeger et al. 2013).

b Accession numbers are for the GEO database for *S. macrospora* and *P. confluens*, and for the Array Express database for *S. pombe*.

The strain used for the RNA-seq analysis was identical to the one used for genome sequencing in the case of *P. confluens* (Traeger et al. 2013). For *S. macrospora*, the wild type strain used for RNA-seq was a derivative of the wild type strain used for the original genome sequencing (Nowrousian et al. 2010); however the genome version (v02) used in our analysis is based on corrections of the original genome sequence based on resequencing of the strain also used for RNA-seq (Nowrousian et al. 2012, Teichert et al. 2012). Therefore, strain differences should not contribute significantly to differences between the RNA-seq data and the genome sequences of the two species. However, to check to what degree such differences or errors in the reference genome sequences contributed to the identified putative editing sites, we analyzed how many putative editing sites occurred in the RNA-seq data from all samples with at least 95% of the variant base. This resulted in 15 sites for *S. macrospora* and 13 sites for *P. confluens*. Thus, the large majority of the hundreds to thousands of potential editing sites that were identified (see below) are not due to strain differences or errors in the genome sequences.

For a comparison of editing sites of *S. macrospora* with *P. confluens* and *F. graminearum*, respectively, orthologs between *S. macrospora* and the other two fungi were identified by reciprocal BLAST as described (Altschul et al. 1997, Traeger et al. 2013). Putative editing sites identified in wild type protoperithecial samples from *S. macrospora* were compared with *F. graminearum* editing sites found by Liu et al. (2016) and with putative editing sites identified in sexual tissue from *P. confluens*. Functional classification of genes with putative editing sites was done using FungiFun 2 (Priebe et al. 2015) with the FunCat ontology (Ruepp et al. 2004), or Ontologizer (Bauer et al. 2008) based on gene ontology (GO) annotations from UniProt (Ashburner et al. 2000, Huntley et al. 2015).

### Verification of RNA Editing Sites by PCR and Sanger Sequencing

To verify that the observed variants are indeed editing sites and not errors in the genome sequences or RNA-seq artefacts, polymerase chain reaction (PCR) fragments from five and six genes covering 12 and 16 putative editing sites of *S. macrospora* and *P. confluens*, respectively, were amplified from genomic DNA as well as cDNA derived from samples grown under conditions for sexual development. Oligonucleotides for PCR are given in supplementary table S1, Supplementary Material online. PCR fragments were either sequenced directly by Sanger sequencing, or cloned into vectors pDrive (Qiagen, Hilden, Germany) or pJet12 (Thermo Fisher Scientific, Waltham, MA, USA) and sequenced.

## Results

### A-to-I RNA Editing in *S. macrospora* Occurs in Young Fruiting Bodies of the Wild Type, but Not in Two Developmental Mutants

**Fig. 1.—:**
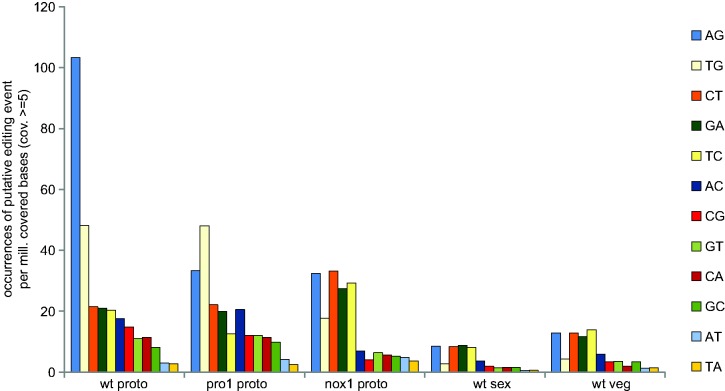
Analysis of putative RNA editing events in *Sordaria macrospora*. RNA-seq data from five conditions or strains were analyzed (Teichert et al. 2012, Dirschnabel et al. 2014). The occurrence of base changes in annotated genes compared with genomic DNA is given as putative RNA editing events per million covered bases, the coverage threshold was set to ≥5. Only base changes detected in two independent samples for each condition were counted. wt = wild type, nox1 and pro1 denote the corresponding mutants; proto, protoperithecia (young fruiting bodies); sex, total sexual mycelium; veg, total vegetative mycelium.

In a recent study on *F. graminearum*, it was found that A-to-I RNA editing occurs specifically in fruiting bodies, but not in vegetative hyphae and conidia (Liu et al. 2016). To analyze whether A-to-I RNA editing also occurs during sexual development of the Sordariomycete *S. macrospora*, and if it is dependent on progression through the protoperithecial stage, we analyzed RNA-seq data from different developmental stages from previous studies. These included data from protoperithecia of the wild type and two developmental mutants as well as wild type total vegetative and sexual mycelium (Teichert et al. 2012, Dirschnabel et al. 2014) (table 1). The vegetative mycelium samples were grown under conditions that do not allow any sexual development (shaking cultures, as *S. macrospora* does not form fruiting bodies when grown submerged). The sexual mycelium samples were grown as surface cultures that induce fruiting body formation and therefore contain protoperithecia; however, the bulk of the harvested tissue consists of vegetative hyphae that do not themselves participate in fruiting body formation. For the protoperithecia samples, young fruiting bodies were separated from the surrounding nonsexual hyphae by laser microdissection. Therefore these samples consist only of hyphae that participate in the formation of fruiting body structures and asci, but do not contain mature asci. Under the applied thresholds (see Materials and Methods), a total of 3848 positions occurring in at least one of the conditions or strains were identified where a nucleotide base in the transcript differed from the genome sequence (see supplementary table S2, Supplementary Material online). The 12 possible nucleotide exchanges were distributed mostly evenly and at low frequency in the total mycelial samples, whereas a slight increase in potential editing events was observed for several possible changes in protoperithecia (fig. 1). However, a strong increase was observed only in wild type protoperithecia, and here only for A-to-G changes (fig. 1). In wild type protoperithecia, 36% of the observed changes are A-to-G changes, whereas A-to-G changes make up less than 20% in the other samples (see supplementary fig. S1, Supplementary Material online), and of the 481 observed A-to-G changes in wild type protoperithecia, 410 occurred only in this condition and not in the other samples (see supplementary table S2, Supplementary Material online, sheet 2). This indicates that in *S. macrospora* A-to-I RNA editing might be present and might preferably occur during sexual development. Interestingly, increase in A-to-G changes in the transcript sequences of developmental mutants pro1 and nox1 was not elevated above the background of potential base changes (fig. 1). Both mutants have a block at the stage of protoperithecia formation and thereby at an early stage of sexual development before maturation of the fruiting body. Therefore, both mutants never produce mature asci or ascospores, and thus are sterile (Masloff et al. 1999, Dirschnabel et al. 2014). This suggests that development has to progress past certain points for A-to-I editing to occur at wild type levels, additionally confirming a correlation with (later) sexual stages that was also observed in *F. graminearum* (Liu et al. 2016).

**Fig. 2.—:**
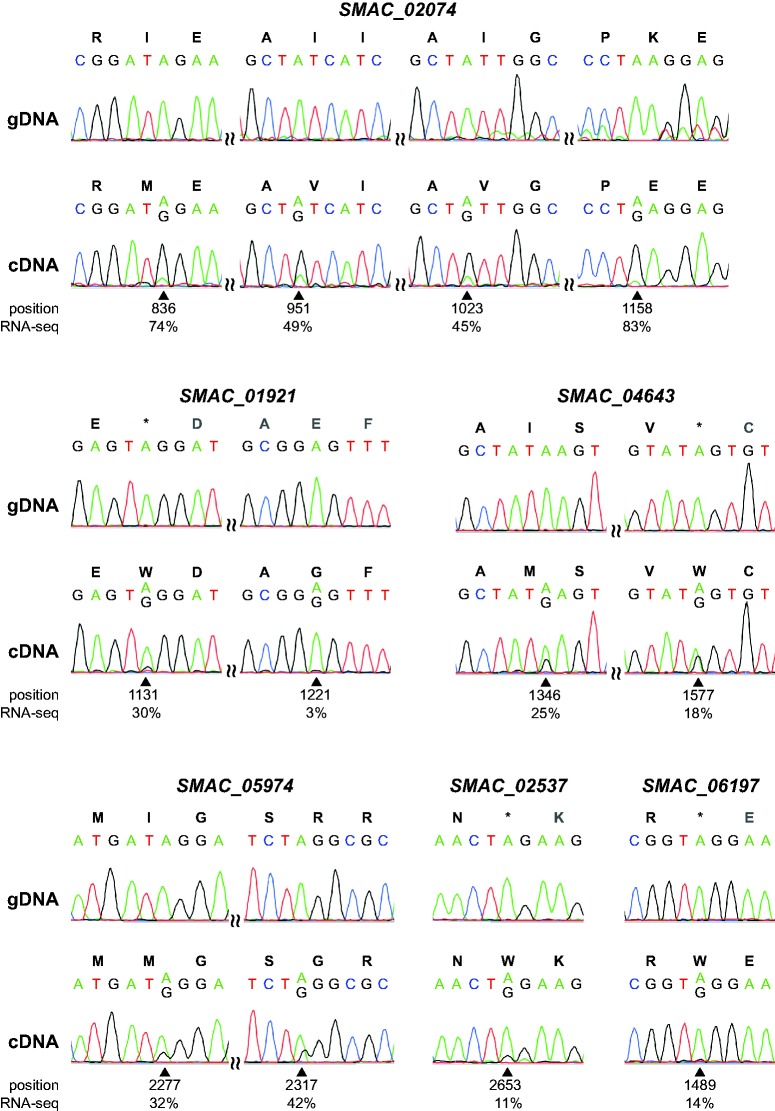
Verification of selected A-to-I RNA editing sites in the *S. macrospora* wild type. PCR fragments derived from genomic DNA (gDNA) or cDNA from samples grown for 6 d under conditions allowing sexual development were sequenced by Sanger sequencing. Chromatograms from sequences around edited sites are shown for gDNA and cDNA of 12 editing sites in six genes. Below each site, the position within the gene and the percentage of edited sequence reads in the RNA-seq data from wild type protoperithecia are given.

**Fig. 3.—:**
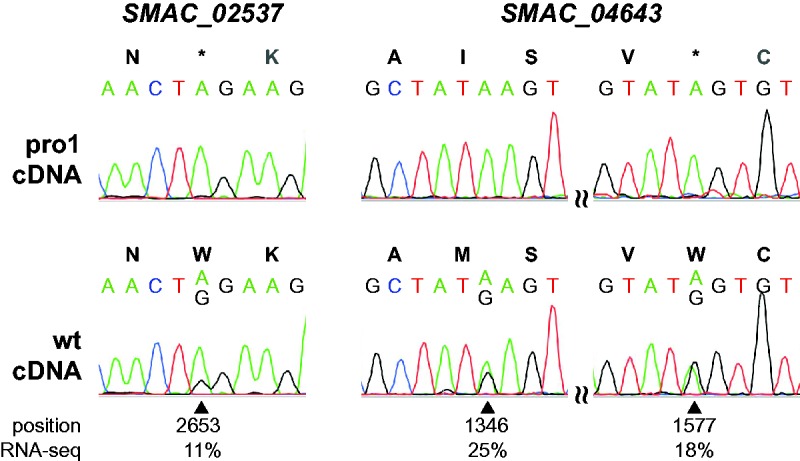
A-to-I editing in the genes *SMAC_02537* and *SMAC_04643* can be detected in the wild type, but not in developmental mutant pro1. PCR fragments derived from cDNA from samples grown for 5d under conditions allowing sexual development were sequenced by Sanger sequencing. Chromatograms from sequences around edited sites are shown for wild type and developmental mutant pro1. Below each site, the position within the gene and the percentage of edited sequence reads in the RNA-seq data from wild type protoperithecia are given. Editing of these sites was not observed in RNA-seq data from pro1 protoperithecia.

To exclude errors introduced by RNA-seq or in the published genome sequence (Nowrousian et al. 2010, Teichert et al. 2012), 12 putative A-to-I editing sites were chosen for verification by Sanger sequencing. For all 12 sites, genomic DNA was sequenced and found to be as expected (fig. 2). cDNA was generated by RT-PCR from the wild type grown under conditions that allow sexual development, and directly sequenced by Sanger sequencing. Samples were grown for 5-6 d, which was longer than the growth time used for the RNA-seq analyses (Teichert et al. 2012), to obtain a higher percentage of sexual tissues in form of larger, more mature fruiting bodies in the samples. With the exception of one site that had a low percentage of editing in the RNA-seq data (position 1489 in gene *SMAC_06197*), the presence of the A-to-G change was confirmed in all cases (fig. 2). For three sites in two genes, editing was also analyzed in mutant pro1 (fig. 3). In contrast to wild type, no base changes were observed in mutant cDNA, verifying the reduction in editing that was observed in the RNA-seq data for the mutant strains.

The fact that we were able to verify most of the chosen A-to-I editing sites that were analyzed in detail indicates that the chosen thresholds and analysis of replicate samples give a low number of false positives. To analyze potential stochasticity in the editing process, we compared the occurrence of potential editing events in each independent replicate with those present in both replicates (table 2). When analyzing all potential editing events, only 5–19% of putative editing events in the independent replicates occur in both replicates. For the A-to-G changes, the numbers are 11–23%, and in both cases, the highest percentage of reproducible putative editing events is found in the wild type protoperithecia. This is consistent with certain stochasticity in the editing phenomenon in general, but suggests higher specificity in the A-to-I editing during sexual development. The 23% reproducible A-to-G changes in wild type protoperithecia is much lower than what was observed for *P. confluens* (see below). However, this might be due to technical issues, specifically a 3' bias in the protoperithecia samples, because these were prepared by laser microdissection and subsequent linear RNA amplification, which leads to overrepresentation of sequences at the 3' end of mRNAs (Teichert et al. 2012).

**Table 2:** Editing Sites in Replicate Experiments with *Sordaria macrospora*

		Normalized Number of Occurences for Putative Editing Event							
		AG	AG	AG	AG	All	All	All	All
Condition	Covered Bases^a^	Rep. 1	Rep. 2	Both	% Both^b^	Rep. 1	Rep. 2	Both	% Both^b^
veg	14022957	84.1	80.6	12.9	15.7	549.2	1148.7	76.7	9.0
sex	12261402	92.3	62.2	8.5	11.0	730.5	1192.1	48.0	5.0
wt proto	4708650	556.5	336.9	103.2	23.1	1621.3	1336.5	283.1	19.1
pro1 proto	4469196	232.7	226.4	33.3	14.5	2008.2	2153.5	208.8	10.0
nox1 proto	6733663	266.6	233.4	32.5	13.0	1931.4	1611.5	177.0	10.0

The number of occurrences (normalized to million covered bases with coverage ≥5) for A-to-G base changes (AG) or all base changes is given for the two independent replicates (rep. 1 and rep. 2) and for base changes that were found in both replicates (both).

a Mean of independent replicates 1 and 2.

b Both as % of mean of independent replicates 1 and 2.

Among the 410 A-to-I editing sites observed only in wild type protoperithecia, 244 are located within coding regions. Of these, 47 would change a stop codon to an amino acid encoding codon (stop-loss change), thereby leading to an extension of the respective protein. Another 172 would lead to a nonsynonymous codon change. Thus, the majority (53%) of A-to-I editing sites observed in wild type protoperithecia are predicted to lead to a different gene product than the one encoded in the genomic DNA (see supplementary table S2, Supplementary Material online).

### A-to-I RNA Editing Occurs during Sexual Development in a Member of an Early-Diverging Lineage of Filamentous Ascomycetes

**Fig. 4.—:**
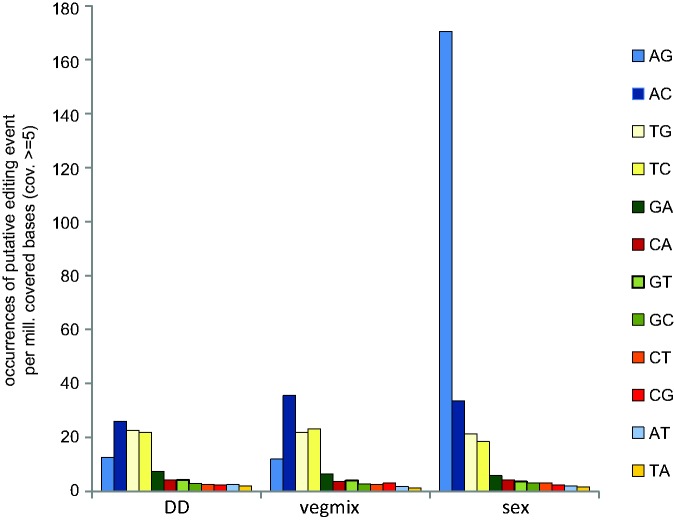
Analysis of putative RNA editing events in *P. confluens*. RNA-seq data from three conditions were analyzed (Traeger et al. 2013). The occurrence of base changes in annotated genes compared with genomic DNA is given as putative RNA editing events per million covered bases, the coverage threshold was set to ≥5. Only base changes detected in two independent samples for each condition were counted. Abbreviations: DD, growth in darkness (*P. confluens* does not form fruiting bodies without light); vegmix, pooled RNA from growth in light but under conditions that do not allow fruiting body formation (submerged growth or growth on complete medium); sex, growth under conditions that allow fruiting body formation (surface cultures in minimal medium in the light).

Previous analyses of A-to-I editing in filamentous ascomycetes were performed with members of the Sordariomycetes (Liu et al. 2016), and the analysis of this fungal group is extended in our data for *S. macrospora*. However, it was not clear if similar editing events might occur also in other groups of filamentous ascoymcetes. Therefore, we analyzed RNA-seq data from a previous study of the Pezizomycete *P. confluens* (Traeger et al. 2013). In this case, two conditions (DD and vegmix) that allow only vegetative hyphal growth were compared with a growth condition that induces sexual development (sex, table 1). In the case of *P. confluens*, the sexual mycelium samples were grown in surface culture in the light, but in contrast to *S. macrospora*, the ratio of developing fruiting bodies to the surrounding vegetative hyphae is much higher in this species. Therefore, the bulk of the sampled mycelium from the sexual cultures of *P. confluens* consists of hyphae that participate in fruiting body formation, whereas only vegetative hyphae are formed in conditions DD and vegmix. Under the same thresholds that were used for *S. macrospora*, a total of 7035 positions occurring in at least one of the conditions tested were identified as putative editing sites in *P. confluens* (see supplementary table S3, Supplementary Material online). The 12 possible nucleotide exchanges were distributed more or less evenly and at low frequencies in the analyzed conditions, except for a strong preference for A-to-G changes in sexual development (fig. 4). Sixty three percent of the observed base changes are A-to-G changes during sexual development, but less than 12% are A-to-G changes in either the DD or the vegmix sample (see supplementary fig. S1, Supplementary Material online). Of the 2841 A-to-G changes observed in sexual development, 2772 occurred only in this condition and not in DD or vegmix samples (see supplementary table S3, Supplementary Material online). This indicates that A-to-I RNA editing might be present in *P. confluens*, and thus in an early-diverging group of filamentous ascomycetes that shared a last common ancestor with the higher filamentous ascomycetes including the Sordariomycetes at least 400 million years ago (Traeger et al. 2013). Furthermore, A-to-I RNA editing seems to be correlated with sexual development in this group of fungi, too, suggesting that this is a conserved feature during fruiting body development in filamentous ascomycetes.

**Fig. 5.—:**
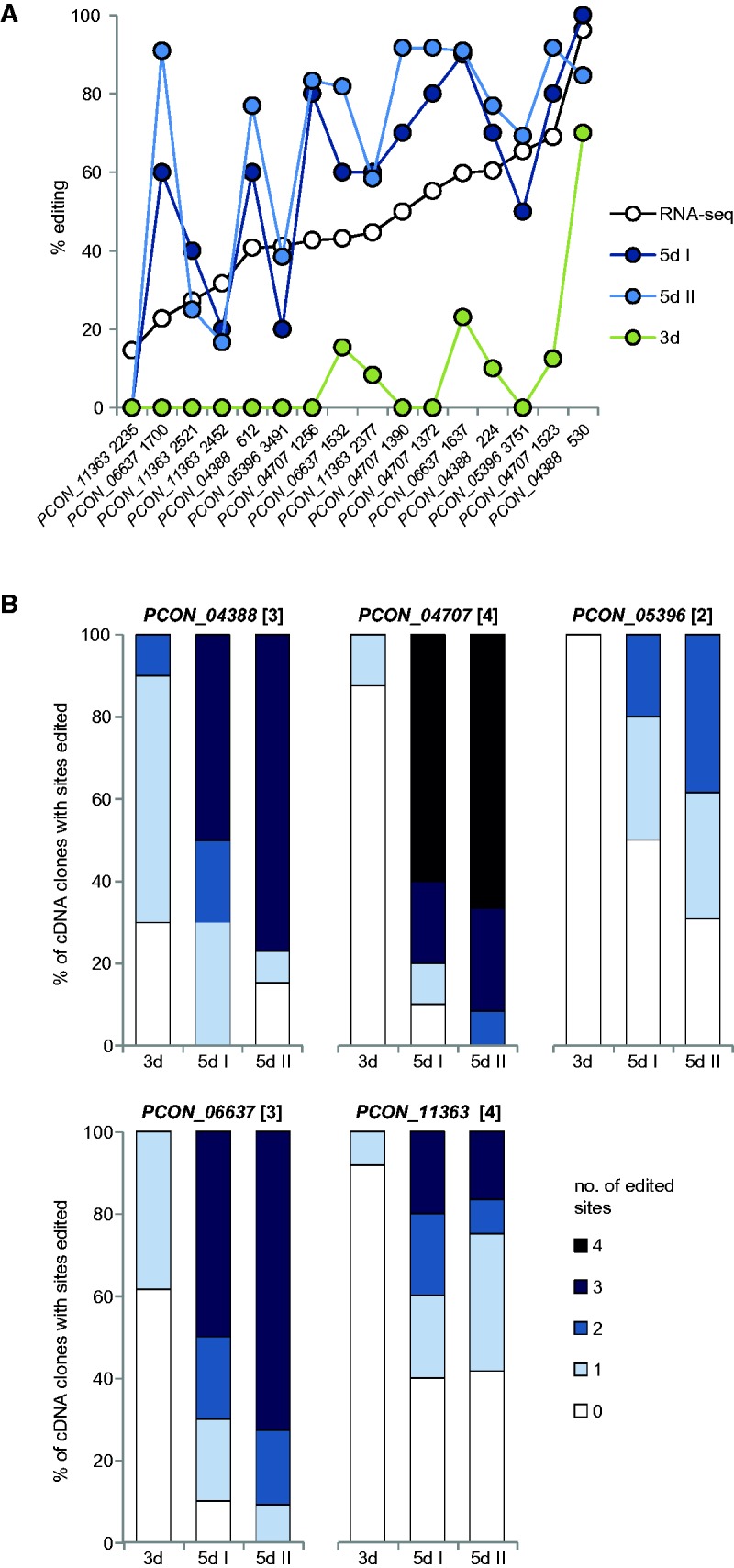
Verification of RNA editing in *P. confluens*. Sixteen putative editing sites in five genes were analyzed by sequencing cDNA clones from sexually developing samples after 3d or 5d (8–14 cDNA clones for each sample). Genomic DNA was also sequenced and was found to be as expected in all cases (data not shown). (*A*) Editing sites were sorted from low to high editing frequency in the RNA-seq data, and the percentage of editing observed in the sequenced cDNA clones in the different samples is indicated for each site. (*B*) For each analyzed gene, the percentage of cDNAs with the indicated number of edited sites is shown for each of the conditions. The number of potential editing sites for each analyzed DNA fragment is given in square brackets after the gene name.

As for *S. macrospora*, we performed verification of several A-to-I editing sites in *P. confluens* to exclude errors in the genome sequence or the RNA-seq data. PCR fragments covering 16 putative A-to-I editing sites from genomic DNA and cDNA were cloned and Sanger sequenced (fig. 5, see supplementary fig. S2, Supplementary Material online). Fifteen sites could be verified; the only site that could not be verified (position 2235 in gene *PCON_11363*) is the one with the lowest percentage of variant base coverage in the RNA-seq data, so it might be edited at rather low frequency. In general, higher editing frequencies in the RNA-seq data correlated with higher frequencies in the sequenced cDNA clones (fig. 5). By sequencing PCR fragments from cDNAs containing several editing sites, we were also able to address the question whether all or only a subset of sites are edited in a single transcript. The analysis of five genes with 2–4 editing sites each showed that at the beginning of sexual development (3d samples in fig. 5B, see supplementary fig. S2, Supplementary Material online) transcripts are incompletely edited or unedited, whereas in later stages (5d samples in fig. 5B, see supplementary fig. S2, Supplementary Material online) transcripts are mostly or completely edited. However, in transcripts with incomplete editing, many different combinations of edited sites were observed (see supplementary fig. S2, Supplementary Material online). Thus, editing sites might be processed independently for each site even within the same RNA molecule, and editing efficiency increases as sexual development progresses.

Similar to the analysis in *S. macrospora*, we compared the occurrence of potential editing events in each independent replicate with those present in both replicates (table 3). In *P. confluens*, between 10 and 22% of all potential editing sites were identified in both replicates, and this number was elevated to 47% specifically for A-to-G changes in sexual samples. Thus, the general trend of higher reproducibility for A-to-G changes in sexual samples is present in both *P. confluens* and *S. macrospora*, and suggests specificity in the A-to-I editing during sexual development. Among the 2772 A-to-I editing sites observed only in sexual development in *P. confluens*, 2592 are located within coding regions, with 149 leading to a stop-loss change and 2175 leading to a nonsynonymous codon change (see supplementary table S3, Supplementary Material online). Thus, similar to the findings in *S. macrospora*, the majority (84%) of A-to-I editing sites in sexual development are predicted to lead to a different gene product compared to the one encoded in the genomic DNA of *P. confluens*.

**Table 3:** Editing Sites in Replicate Experiments with *Pyronema confluens*

		Normalized Number of Occurences for Putative Editing Event							
		AG	AG	AG	AG	All	All	All	All
Condition	Covered Bases^a^	Rep. 1	Rep. 2	Both	% Both^b^	Rep. 1	Rep. 2	Both	% Both^b^
DD	13956949	193.9	151.8	12.6	7.3	1277.0	1014.1	111.9	9.8
vegmix	14873894	89.8	159.8	12.0	9.6	693.9	1103.4	119.1	13.3
sex	16733099	293.3	424.4	170.5	47.5	1041.6	1407.7	270.5	22.1

The number of occurrences (normalized to million covered bases with coverage ≥5) for A-to-G base changes (AG) or all base changes is given for the two independent replicates (rep. 1 and rep. 2) and for base changes that were found in both replicates (both).

a Mean of independent replicates 1 and 2.

b Both as % of mean of independent replicates 1 and 2.

### Individual A-to-I RNA Editing Sites Are Not Conserved Between Different Ascomycetes

As the process of A-to-I editing might be conserved in different groups of filamentous ascomycetes, we wondered if this is also true for editing of orthologous genes or even individual editing sites. In *F. graminearum*, Liu et al. (2016) focused on editing events that led to the loss of a stop codon and therefore extension of the corresponding protein (stop-loss events), and on stop-loss events specifically in sequences that were erroneously annotated as introns, also leading to longer and different protein sequences (*puk1*-like editing) (Liu et al. 2016). We therefore analyzed whether edited genes identified to undergo stop-loss or *puk1*-like editing in *F. graminearum* were also among the A-to-I edited genes in wild type protoperithecia in *S. macrospora* (see supplementary table S4, Supplementary Material online). Of the 240 genes undergoing stop-loss or *puk1*-like editing in *F. graminearum* and having an orthologous gene in *S. macrospora*, only nine genes also undergo editing in the *S. macrospora* wild type protoperithecia.

We also analyzed which orthologous genes are edited in *S. macrospora* and *P. confluens* for those cases where editing leads to an amino acid change and the editing site in the corresponding gene has at least 10% of edited bases in the RNA-seq data. This resulted in 24 editing sites in 21 genes (see supplementary table S5, Supplementary Material online). However, closer inspection of those sites that lead to the same amino acid change in the orthologous proteins showed that in no case was this change at an orthologous amino acid position, suggesting that editing of these sites evolved independently (data not shown). Overall, we did not observe a high degree of conservation of A-to-I editing sites between *S. macrospora* and either the distantly related *P. confluens* or the more closely related *F. graminearum*. This suggests that while A-to-I editing during sexual development might be a conserved process in filamentous ascomycetes, individual editing sites can undergo comparatively rapid evolutionary turnover. An alternative hypothesis would be that A-to-I editing evolved independently in different species, which might explain the lack of conserved orthologous editing sites.

**Fig. 6.—:**
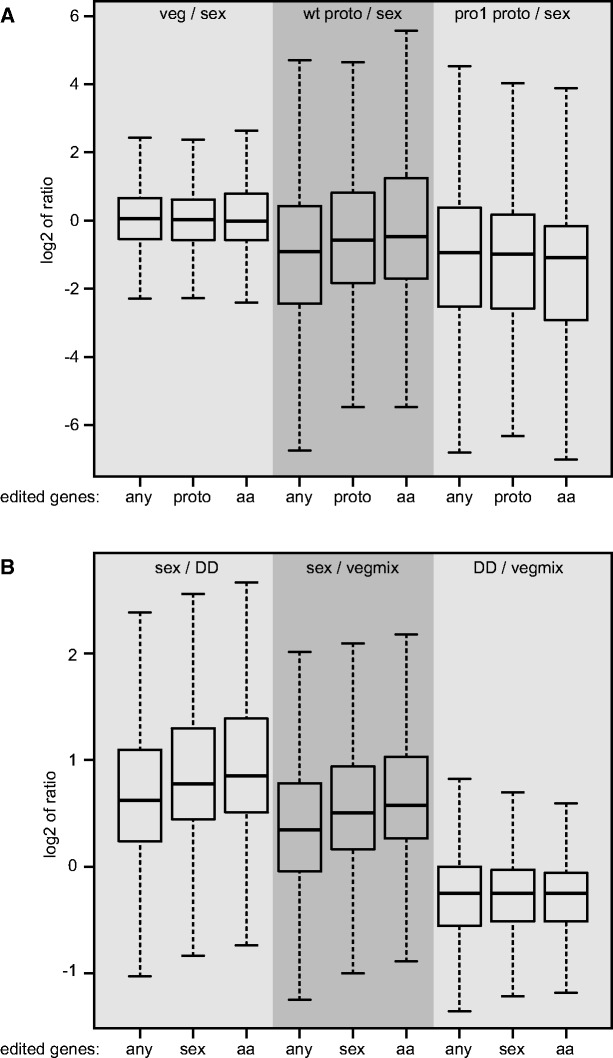
Analysis of expression of edited genes in *S. macrospora* and *P. confluens*. Boxplots showing the distribution of expression ratios of edited genes (outliers left out for better visibility) with the median value as a horizontal line in the box between the first and third quartiles. (*A*) *Sordaria macrospora* genes with any type of putative editing site (any), genes with A-to-I editing only in wild type protoperithecia (proto), and genes with A-to-I editing only in wild type protoperithecia with editing in at least 10% of observed transcripts and where editing leads to an amino acid change (aa) were analyzed for expression in three different conditions based on published RNA-seq data (Teichert et al. 2012). The conditions analyzed are expression ratios of vegetative vs. sexual mycelium (veg/sex), wild type protoperithecia vs. sexual mycelium (wt proto/sex), and pro1 protoperithecia vs. sexual mycelium (pro1 proto/sex). (*B*) *Pyronema confluens* genes with any type of putative editing site (any), genes with A-to-I editing only in sexual mycelium (sex), and genes with A-to-I editing only in sexual mycelium with editing in at least 10% of observed transcripts and where editing leads to an amino acid change (aa) were analyzed for expression in three different conditions based on published RNA-seq data (Traeger et al. 2013). The conditions analyzed are expression ratios of sexual mycelium vs. vegetative mycelium grown in darkness (sex/DD), sexual mycelium vs. vegetative mycelium from several growth conditions (sex/vegmix), and a comparison of the two different growth conditions yielding only vegetative mycelium (DD/vegmix).

To analyze if there are general trends in function or expression of edited genes in different groups of fungi, functional classification and transcript levels of genes with editing sites were analyzed. Functional classification of genes that show A-to-I editing only in wild type protoperithecia of *S. macrospora* (see supplementary fig. S3, Supplementary Material online) or sexual mycelium of *P. confluens* (see supplementary fig. S4, Supplementary Material online) revealed a number of cellular functions that might be influenced by the edited genes. However, due to the fact that only about 400 genes were analyzed for *S. macrospora* (compared to about 2700 genes for *P. confluens*), few significantly enriched functional categories could be identified in *S. macrospora*, and a direct comparison of the results is difficult. When comparing the expression of edited genes, similar trends were observed in *S. macrospora* and *P. confluens* (fig. 6). In both species, median expression ratios in comparisons of sexual tissue vs. vegetative mycelium increased in groups of genes that show A-to-I editing during sexual development compared with groups of genes that contain any putative editing sites in at least one of the analyzed conditions (wt proto/sex in fig. 6A, sex/DD and sex/vegmix in fig. 6B). This increase is even more pronounced in groups of genes that show A-to-I editing during sexual development leading to an amino acid change in the predicted protein. In conditions that did not lead to an observed increase in A-to-I editing (comparisons veg/sex and pro1 proto/sex in *S. macrospora*, and DD/vegmix in *P. confluens*), no differences in overall expression levels are observed between the different groups of edited genes. Thus, the data show a correlation between A-to-I editing that leads to changes in protein sequences, and therefore possibly to functional changes, and an increase in gene expression during sexual development in *S. macrospora* and *P. confluens*.

### A-to-I RNA Editing Was Not Detected in *S. pombe*

The finding that A-to-I RNA editing seems to be a conserved feature during sexual development throughout filamentous ascomycetes made us wonder if this phenomenon is present in the earlier-diverging groups of ascomycetes that, with few exceptions, do not form fruiting bodies during sexual development. Therefore, we analyzed a publicly available RNA-seq dataset from samples of different meiotic stages of the Taphrinomycete *S. pombe* (Wilhelm et al. 2008). The analyzed samples include five different meiotic stages and one vegetatively grown sample (table 1). There was a strong C-to-T increase during sexual development in *S. pombe* (meiotic samples); however, in contrast to *S. macrospora* and *P. confluens*, there was no preference for A-to-G changes (see supplementary fig. S5 and table S6, Supplementary Material online). Based on this analysis, we currently cannot conclude that A-to-I editing is present in *S. pombe*. It might be that the sexual development-related A-to-I RNA editing evolved only after the split of Taphrinomycetes from the ancestor of other ascomycete groups. Alternatively, the lineage leading to *S. pombe* might have lost this type of editing during the evolution to a unicellular growth form. However, we currently cannot exclude that A-to-I editing is present but has not been detected in the analyzed samples due to low coverage in the available dataset. Analysis of RNA-seq samples from additional Taphrinomycotina as well as Saccharomycotina will be necessary to address the question of the evolution of A-to-I RNA editing in ascomycetes.

## Discussion

In this study, we analyzed RNA-seq data from *S. macrospora*, *P. confluens*, and *S. pombe* for the presence of putative RNA editing events. A-to-I RNA editing was found to be present during sexual development in the filamentous ascomycetes *S. macrospora* and *P. confluens*, but not in the Taphrinomycete *S. pombe*. A number of editing sites were confirmed independently by Sanger sequencing in *S. macrospora* and *P. confluens*. Sequencing of cloned cDNAs in *P. confluens* showed that editing of several sites within a single transcript can occur independently and that editing penetrance increases during progression of sexual development. Analysis of editing in two developmental mutants of *S. macrospora* indicated that sexual development has to progress through certain stages for editing to occur at significant levels.

Our data suggest that sexual development-dependent A-to-I RNA editing is conserved in filamentous ascomycetes, even though this does not extend to individual RNA editing sites as indicated by a comparison between *S. macrospora* and *P. confluens*. Similarly, a comparison of *S. macrospora* and the more closely related *F. graminearum* showed that the 240 orthologous genes that undergo stop-loss or puk1-like editing in *F. graminearum* (Liu et al. 2016) are rarely edited in *S. macrospora*. However, this is in line with the finding by Liu and coworkers concerning editing of the *puk1* gene in *F. graminearum*, *F. verticilloides*, and *N. crassa*. Of the two stop codons edited in *F. graminearum puk1*, only the first is conserved at sequence and editing levels in *N. crassa*, and only the second in *F. verticilloides* (Liu et al. 2016). Thus, the process of A-to-I editing during sexual development might be conserved in filamentous ascomycetes, but individual editing sites are not necessarily conserved. In the case of *puk1*, editing might be present in those cases where a mutation resulted in a stop codon that would otherwise render the proteins nonfunctional, consistent with a hypothesis of RNA editing evolving under a model of neutral evolution (Gray 2012). Given the large evolutionary distance between the Sordariomycetes and Pezizomycetes, an alternative hypothesis explaining the lack of conservation of individual editing sites would be that editing during sexual development evolved independently in the Sordaria/Fusarium and Pyronema lineages. However, fast evolution of A-to-I editing sites was also found in metazoa, for example in the caste-specific A-to-I editing in leaf-cutting, fungal-growing ants, where editing sites seem to be mostly species-specific and fast-evolving (Li et al. 2014). Even editing of highly conserved positions in the calcium sensor synaptotagmin I in the insect nervous systems evolved within 250 million years of insect evolution to vary from no editing to the editing-dependent change of up to four amino acids (Reenan 2005). In mammals, some editing sites are conserved, but the majority is not conserved and edited at low frequencies. In the analyzed cases, most of the conserved sites are present in coding regions, lead to codon changes, and tend to result in an ancient version of the encoded protein (Pinto et al. 2014).

For the lack of observed A-to-I editing events in *S. pombe* there are several possible explanations, including the evolution of A-to-I editing after the split of the Taphrinomycotina from the other ascomycetes or the loss of editing in yeasts. However, additional data with higher sequence read coverage and from more species from early-diverging ascoymcete lineages are needed to address the question of the evolution of A-to-I RNA editing in ascomycetes. With respect to basidiomycetes, there is only one study in *G. lucidum* where editing was analyzed in fruiting body samples; however, no preference for A-to-I editing was found (Zhu et al. 2014). This might suggest that sexual development-specific A-to-I editing evolved in the ascomycetes, but additional basidiomycete samples from different growth conditions and species would be required to test this hypothesis.

In an analysis of *F. graminearum* fruiting bodies, more than 26,000 A-to-I editing sites were identified (Liu et al. 2016). Here, we observed about 400 and 2700 A-to-I editing sites specific to young fruiting bodies of the *S. macrospora* wild type and sexual development samples of *P. confluens*, respectively. The overall lower numbers of editing sites observed in our study might be caused by differences in the analyzed samples with respect to sexual development. In the *F. graminearum* study, fully mature fruiting bodies were analyzed (Liu et al. 2016), whereas the RNA-seq data analyzed in our study were generated from samples that contain younger stages of total mycelium undergoing sexual development or are from young fruiting bodies well before maturation (Teichert et al. 2012, Traeger et al. 2013). For *S. macrospora*, these data allowed us to compare mutants with a block at the stage of protoperithecium formation with the wild type. This showed that in the mutants, no significant increase in A-to-I editing occurred, whereas editing is found in wild type protoperithecia, indicating that fruiting body development has to progress past certain steps for editing to occur at detectable levels. However, in the immature fruiting bodies of the wild type of both *S. macrospora* and *P. confluens*, one would expect sexual development-related editing not to have reached its maximum. This is confirmed by the Sanger sequencing of cDNA clones from *P. confluens* that showed an overall increase in editing frequency from 3d to 5d sexual growth (fig. 5B). One possibility is that editing occurs preferentially in cell types contributing to spore formation and maturation, for example ascogenous hyphae in later stages of development. Such tissues would be absent or only make up a small part of the analyzed samples during early stages of fruiting body development. In the case of *S. macrospora*, technical limitations due to 3' bias in the laser microdissected protoperithecia samples and consequently sequence coverage of fewer genomic bases could also contribute to the detection of fewer A-to-I editing sites. Another difference of our analysis compared with the analysis of *F. graminearum* is that in the latter study, RNA-seq data from two independent samples were pooled to identify more editing sites through higher coverage, resulting in about 26,000 A-to-I editing sites. When counting editing sites present in both independent samples, this gave about 17,000 editing sites for *F. graminearum* (Liu et al. 2016). In our analysis, we counted putative editing sites present in two independent samples, which led to considerably fewer sites than when counting sites observed in one sample (tables 2 and 3).

The finding that A-to-I RNA editing is associated with sexual development in distantly related filamentous ascomycetes begs the question why RNA editing would evolve to occur during this specific process. A number of hypotheses have been proposed to explain the evolution of RNA editing in general, and these might also be applicable to fungi. One hypothesis is that RNA editing might facilitate adaptive evolution by allowing the parallel existence of multiple gene products from a single genomic locus (Gommans et al. 2009, Rosenthal 2015). Under this model, RNA editing capabilities can also lead to the evolution of increasingly complex systems and organism. This hypothesis might be compatible with the finding that editing in filamentous ascomycetes is tied to sexual development, and thus to the most complex structures that these fungi differentiate. It is also supported by the finding that genes with editing sites that lead to a change in the predicted protein sequence show on average higher expression in sexual development compared with genes with any editing site. Therefore, the genes coding for editing-modified gene products might be increasingly expressed, and possibly involved, in sexual development.

Another model focuses on neutral evolution, that is an RNA editing system would be in place first, then mutations that needed fixing could accumulate due to relaxed constraints through selection (Gray 2012). Such a model could explain why there is so little conservation of individual editing sites, because higher mutational loads would be “masked” by editing; however, it would not necessarily explain why editing is restricted to the sexual stage. One possible explanation for this could be that the genes required for editing are active only during this stage, thereby restricting any “corrections” to sexual development. The neutral evolution model might also explain why there might be certain stochasticity in the RNA editing process as evidenced by the finding that a large number of potential editing sites are present in only one of two independent replicates in *S. macrospora* and *P. confluens* (tables 2 and 3).

At present, the enzymes and regulators necessary for A-to-I editing in filamentous ascomycetes are unknown. In contrast to metazoa, where A-to-I editing is mediated by ADAR enzymes, ascomycete editing must be performed by a different set of proteins, because no ADAR orthologs have been identified in fungi (Jin et al. 2009, Grice and Degnan 2015, Liu et al. 2016). Similar to the analysis in other fungi, searches in the genomes of *S. macrospora* and *P. confluens* identified homologs to ADATs (adenosine deaminase acting on tRNA, see supplementary table S7, Supplementary Material online), but no ADAR homologs. Metazoan ADAR proteins usually comprise an adenosine deaminase (AD) domain and a double-stranded RNA binding (dsRB) domain that are thought to contribute to target specificity (Bass 2002). However, no combined AD/dsRB proteins have been identified in fungi. Therefore, it is possible that fungi evolved their own class of A-to-I editing enzymes. Liu and coworkers put forward the hypothesis that in fungi, ADAT-like instead of ADAR enzymes might be involved in A-to-I editing of mRNAs (Liu et al. 2016). It is also possible that specificity in fungi is not conferred by proteins, as RNA editing enzymes for different editing events in other species are known to rely on small RNAs to confer specificity (Knoop 2011). More insights into the evolution and function of sexual development-related A-to-I editing in filamentous ascomycetes will be possible when factors required for editing during ascomycete sexual development have been identified. Furthermore, future studies will focus on determining changes in localization and function of proteins caused by editing of the encoding transcripts.

## Supplementary Material

Supplementary data are available at *Genome Biology and Evolution* online.

## Acknowledgments

We would like to thank Swenja Ellßel, Ingeborg Godehardt, Regina Krampe, and Silke Nimtz for excellent technical assistance, and the German Research Foundation (DFG, Deutsche Forschungsgemeinschaft) for funding (grants KU517/11-2, KU517/16-1, NO407/5-1, and the Open Access Publication Funds of the Ruhr-Universität Bochum).

## Supplementary Material

Supplementary Data
