# Supplementary material for: RNA Editing During Sexual Development Occurs in Distantly Related Filamentous Ascomycetes
Source: Genome Biol Evol. 2017 Apr 1;9(4):855–68. doi: 10.1093/gbe/evx052 (PMC5381528; doi:10.1093/gbe/evx052)
Supplement: Supplementary Data [file evx052_Supp.zip › evx052_Supp/Supplementary_Figures.pdf]

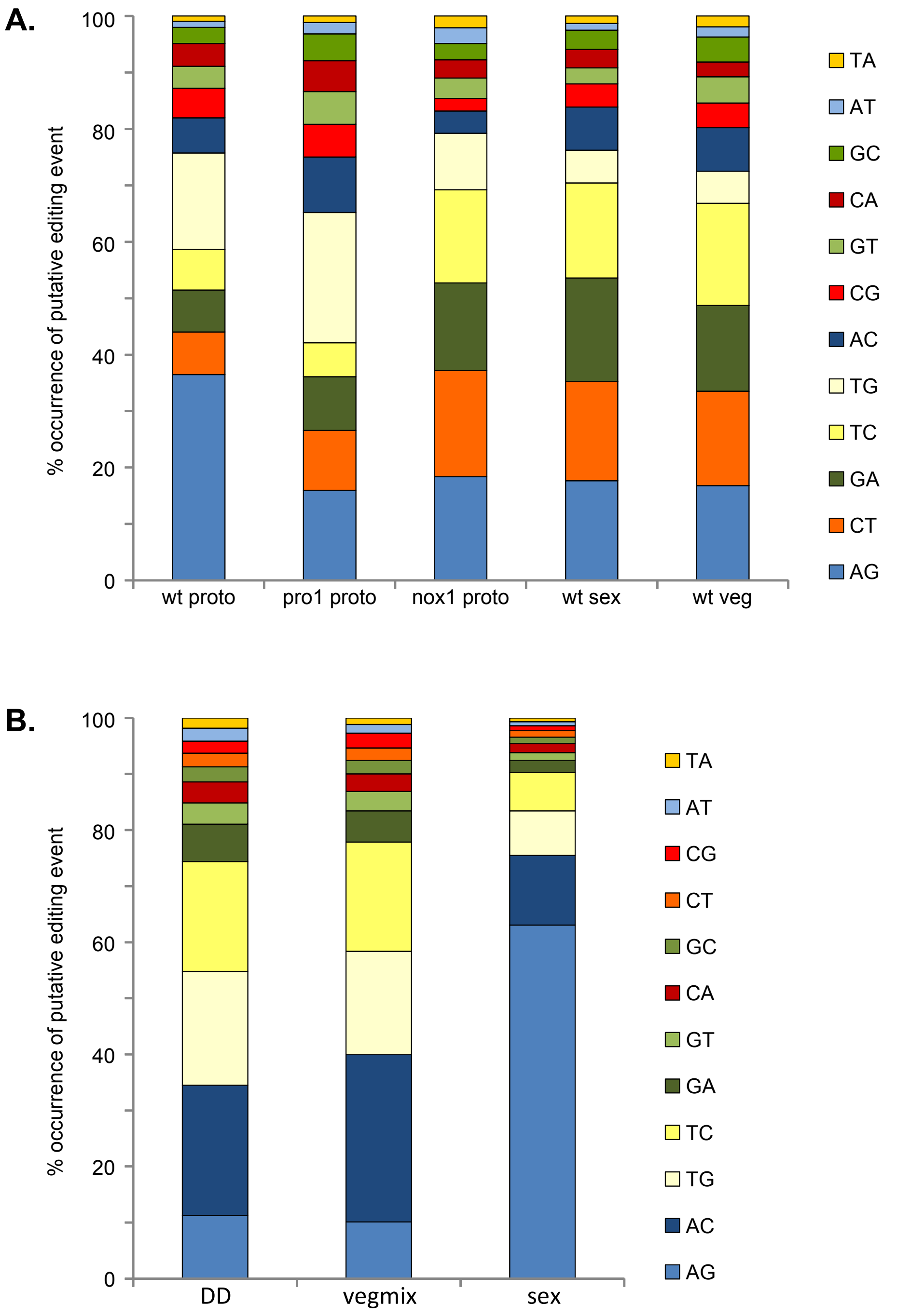


**Figure S1.** Analysis of putative editing events in RNA-seq data from *S. macrospora* (A) and *P. confluens* (B). Relative occurrence of base changes in the analyzed conditions is given in percent.

## Supplementary Figure S2 (parts A-E, legend below part E)

### A. *PCON_04388*

```
                 224            530            612
RNA-seq          60%            96%            40%
                  |              |              |
PCON_04388 ACCTTATTGAC...GCCATAAAACG...CACATAAGCTC
----------------------------------------------------
5d LL I           |              |              |
cDNA1      ACCTTATTGAC...GCCATGAAACG...CACATAAGCTC
cDNA2      ACCTTATTGAC...GCCATGAAACG...CACATAAGCTC
cDNA3      ACCTTATTGAC...GCCATGAAACG...CACATGAGCTC
cDNA4      ACCTTGTTGAC...GCCATGAAACG...CACATAAGCTC
cDNA5      ACCTTGTTGAC...GCCATGAAACG...CACATAAGCTC
cDNA6      ACCTTGTTGAC...GCCATGAAACG...CACATGAGCTC
cDNA7      ACCTTGTTGAC...GCCATGAAACG...CACATGAGCTC
cDNA8      ACCTTGTTGAC...GCCATGAAACG...CACATGAGCTC
cDNA9      ACCTTGTTGAC...GCCATGAAACG...CACATGAGCTC
cDNA10     ACCTTGTTGAC...GCCATGAAACG...CACATGAGCTC
----------------------------------------------------
5d LL II          |              |              |
cDNA1      ACCTTATTGAC...GCCATAAAACG...CACATAAGCTC
cDNA2      ACCTTATTGAC...GCCATAAAACG...CACATAAGCTC
cDNA3      ACCTTATTGAC...GCCATGAAACG...CACATAAGCTC
cDNA4      ACCTTGTTGAC...GCCATGAAACG...CACATGAGCTC
cDNA5      ACCTTGTTGAC...GCCATGAAACG...CACATGAGCTC
cDNA6      ACCTTGTTGAC...GCCATGAAACG...CACATGAGCTC
cDNA7      ACCTTGTTGAC...GCCATGAAACG...CACATGAGCTC
cDNA8      ACCTTGTTGAC...GCCATGAAACG...CACATGAGCTC
cDNA9      ACCTTGTTGAC...GCCATGAAACG...CACATGAGCTC
cDNA10     ACCTTGTTGAC...GCCATGAAACG...CACATGAGCTC
cDNA11     ACCTTGTTGAC...GCCATGAAACG...CACATGAGCTC
cDNA12     ACCTTGTTGAC...GCCATGAAACG...CACATGAGCTC
cDNA13     ACCTTGTTGAC...GCCATGAAACG...CACATGAGCTC
----------------------------------------------------
3d LL II          |              |              |
cDNA1      ACCTTATTGAC...GCCATAAAACG...CACATAAGCTC
cDNA2      ACCTTATTGAC...GCCATAAAACG...CACATAAGCTC
cDNA3      ACCTTATTGAC...GCCATAAAACG...CACATAAGCTC
cDNA4      ACCTTATTGAC...GCCATGAAACG...CACATAAGCTC
cDNA5      ACCTTATTGAC...GCCATGAAACG...CACATAAGCTC
cDNA6      ACCTTATTGAC...GCCATGAAACG...CACATAAGCTC
cDNA7      ACCTTATTGAC...GCCATGAAACG...CACATAAGCTC
cDNA8      ACCTTATTGAC...GCCATGAAACG...CACATAAGCTC
cDNA9      ACCTTATTGAC...GCCATGAAACG...CACATAAGCTC
cDNA10     ACCTTGTTGAC...GCCATGAAACG...CACATAAGCTC
```

## B. *PCON_04707*

```
                  1256          1372          1390          1523
RNA-seq            43%           55%           50%           69%
                    |             |             |             |
PCON_04707 GCATTATTGAC...TCGCTAACACC...AAGCTAGAAAC...GCTTTAGAAAC
-----------------------------------------------------------------
5d LL I             |             |             |             |
cDNA1      GCATTATTGAC...TCGCTAACACC...AAGCTAGAAAC...GCTTTAGAAAC
cDNA2      GCATTGTTGAC...TCGCTAACACC...AAGCTAGAAAC...GCTTTAGAAAC
cDNA3      GCATTATTGAC...TCGCTGACACC...AAGCTGGAAAC...GCTTTGGAAAC
cDNA4      GCATTGTTGAC...TCGCTGACACC...AAGCTAGAAAC...GCTTTGGAAAC
cDNA5      GCATTGTTGAC...TCGCTGACACC...AAGCTGGAAAC...GCTTTGGAAAC
cDNA6      GCATTGTTGAC...TCGCTGACACC...AAGCTGGAAAC...GCTTTGGAAAC
cDNA7      GCATTGTTGAC...TCGCTGACACC...AAGCTGGAAAC...GCTTTGGAAAC
cDNA8      GCATTGTTGAC...TCGCTGACACC...AAGCTGGAAAC...GCTTTGGAAAC
cDNA9      GCATTGTTGAC...TCGCTGACACC...AAGCTGGAAAC...GCTTTGGAAAC
cDNA10     GCATTGTTGAC...TCGCTGACACC...AAGCTGGAAAC...GCTTTGGAAAC
-----------------------------------------------------------------
5d LL II            |             |             |             |
cDNA1      GCATTGTTGAC...TCGCTAACACC...AAGCTAGAAAC...GCTTTGGAAAC
cDNA2      GCATTATTGAC...TCGCTGACACC...AAGCTGGAAAC...GCTTTGGAAAC
cDNA3      GCATTATTGAC...TCGCTGACACC...AAGCTGGAAAC...GCTTTGGAAAC
cDNA4      GCATTGTTGAC...TCGCTGACACC...AAGCTGGAAAC...GCTTTAGAAAC
cDNA5      GCATTGTTGAC...TCGCTGACACC...AAGCTGGAAAC...GCTTTGGAAAC
cDNA6      GCATTGTTGAC...TCGCTGACACC...AAGCTGGAAAC...GCTTTGGAAAC
cDNA7      GCATTGTTGAC...TCGCTGACACC...AAGCTGGAAAC...GCTTTGGAAAC
cDNA8      GCATTGTTGAC...TCGCTGACACC...AAGCTGGAAAC...GCTTTGGAAAC
cDNA9      GCATTGTTGAC...TCGCTGACACC...AAGCTGGAAAC...GCTTTGGAAAC
cDNA10     GCATTGTGGAC...TCGCTGACACC...AAGCTGGAAAC...GCTTTGGAAAC
cDNA11     GCATTGTTGAC...TCGCTGACACC...AAGCTGGAAAC...GCTTTGGAAAC
cDNA12     GCATTGTTGAC...TCGCTGACACC...AAGCTGGAAAC...GCTTTGGAAAC
-----------------------------------------------------------------
3d LL II            |             |             |             |
cDNA1      GCATTATTGAC...TCGCTAACACC...AAGCTAGAAAC...GCTTTAGAAAC
cDNA2      GCATTATTGAC...TCGCTAACACC...AAGCTAGAAAC...GCTTTAGAAAC
cDNA3      GCATTATTGAC...TCGCTAACACC...AAGCTAGAAAC...GCTTTAGAAAC
cDNA4      GCATTATTGAC...TCGCTAACACC...AAGCTAGAAAC...GCTTTAGAAAC
cDNA5      GCATTATTGAC...TCGCTAACACC...AAGCTAGAAAC...GCTTTAGAAAC
cDNA6      GCATTATTGAC...TCGCTAACACC...AAGCTAGAAAC...GCTTTAGAAAC
cDNA7      GCATTATTGAC...TCGCTAACACC...AAGCTAGAAAC...GCTTTAGAAAC
cDNA8      GCATTATTGAC...TCGCTAACACC...AAGCTAGAAAC...GCTTTGGAAAC
```

## C. *PCON_05693*

```
                   3491          3751
RNA-seq             41%           65%
                     |             |
PCON_05693 TGCCCAGGAGC...ACGTTAATGCC
------------------------------------
5d LL I              |             |
cDNA1      TGCCCAGGAGC...ACGTTAATGCC
cDNA2      TGCCCAGGAGC...ACGTTAATGCC
cDNA3      TGCCCAGGAGC...ACGTTAATGCC
cDNA4      TGCCCAGGAGC...ACGTTAATGCC
cDNA5      TGCCCAGGAGC...ACGTTAATGCC
cDNA6      TGCCCAGGAGC...ACGTTGATGCC
cDNA7      TGCCCAGGAGC...ACGTTGATGCC
cDNA8      TGCCCAGGAGC...ACGTTGATGCC
cDNA9      TGCCCGGGAGC...ACGTTGATGCC
cDNA10     TGCCCGGGAGC...ACGTTGATGCC
------------------------------------
5d LL II             |             |
cDNA1      TGCCCAGGAGC...ACGTTAATGCC
cDNA2      TGCCCAGGAGC...ACGTTAATGCC
cDNA3      TGCCCAGGAGC...ACGTTAATGCC
cDNA4      TGCCCAGGAGC...ACGTTAATGCC
cDNA5      TGCCCAGGAGT...ACGTTGATGCC
cDNA6      TGCCCAGGAGT...ACGTTGATGCC
cDNA7      TGCCCAGGAGC...ACGTTGATGCC
cDNA8      TGCCCAGGAGC...ACGTTGATGCC
cDNA9      TGCCCGGGAGC...ACGTTGATGCC
cDNA10     TGCCCGGGAGC...ACGTTGATGCC
cDNA11     TGCCCGGGAGC...ACGTTGATGCC
cDNA12     TGCCCGGGAGC...ACGTTGATGCC
cDNA13     TGCCCGGGAGC...ACGTTGATGCC
------------------------------------
3d LL II             |             |
cDNA1      TGCCCAGGAGC...ACGTTAATGCC
cDNA2      TGCCCAGGAGC...ACGTTAATGCC
cDNA3      TGCCCAGGAGC...ACGTTAATGCC
cDNA4      TGCCCAGGAGC...ACGTTAATGCC
cDNA5      TGCCCAGGAGC...ACGTTAATGCC
cDNA6      TGCCCAGGAGC...ACGTTAATGCC
cDNA7      TGCCCAGGAGC...ACGTTAATGCC
cDNA8      TGCCCAGGAGC...ACGTTAATGCC
cDNA9      TGCCCAGGAGC...ACGTTAATGCC
cDNA10     TGCCCAGGAGC...ACGTTAATGCC
cDNA11     TGCCCAGGAGC...ACGTTAATGCC
cDNA12     TGCCCAGGAGC...ACGTTAATGCC
cDNA13     TGCCCAGGAGC...ACGTTAATGCC
cDNA14     TGCCCAGGAGC...ACGTTAATGCC
```

## D. *PCON_06637*

```
                 1532          1637          1700
RNA-seq           43%           60%           23%
                   |             |             |
PCON_06637  TGGATAAGGTC...CGTATAGAATG...TCGGTAGTGGC
--------------------------------------------------
5d LL I            |             |             |
cDNA1       TGGATAAGGTC...CGTATAGAATG...TCGGTAGTGGC
cDNA2       TGGATAAGGTC...CGTATGGAATG...TCGGTAGTGGC
cDNA3       TGGATAAGGTC...CGTATGGAATG...TCGGTAGTGGC
cDNA4       TGGATAAGGTC...CGTATGGAATG...TCGGTGGTGGC
cDNA5       TGGATGAGGTC...CGTATGGAATG...TCGGTAGTGGC
cDNA6       TGGATGAGGTC...CGTATGGAATG...TCGGTGGTGGC
cDNA7       TGGATGAGGTC...CGTATGGAATG...TCGGTGGTGGC
cDNA8       TGGATGAGGTC...CGTATGGAATG...TCGGTGGTGGC
cDNA9       TGGATGAGGTC...CGTATGGAATG...TCGGTGGTGGC
cDNA10      TGGATGAGGTC...CGTATGGAATG...TCGGTGGTGGC
--------------------------------------------------
5d LL II           |             |             |
cDNA1       TGGATAAGGTC...CGTATGGAGTG...TCGGTAGTGGC
cDNA2       TGGATAAGGTC...CGTATGGAGTG...TCGGTGGTGGC
cDNA3       TGGATGAGGTC...CGTATAGAATG...TCGGTGGTGGC
cDNA4       TGGATGAGGTC...CGTATGGAATG...TCGGTGGTGGC
cDNA5       TGGATGAGGTC...CGTATGGAATG...TCGGTGGTGGC
cDNA6       TGGATGAGGTC...CGTATGGAGTG...TCGGTGGTGGC
cDNA7       TGGATGAGGTC...CGTATGGAGTG...TCGGTGGTGGC
cDNA8       TGGATGAGGTC...CGTATGGAGTG...TCGGTGGTGGC
cDNA9       TGGATGAGGTC...CGTATGGAGTG...TCGGTGGTGGC
cDNA10      TGGATGAGGTC...CGTATGGAGTG...TCGGTGGTGGC
cDNA11      TGGATGAGGTC...CGTATGGAGTG...TCGGTGGTGGC
--------------------------------------------------
3d LL II           |             |             |
cDNA1       TGGATAAGGTC...CGTATAGAATG...TCGGTAGTGGC
cDNA2       TGGATAAGGTC...CGTATAGAATG...TCGGTAGTGGC
cDNA3       TGGATAAGGTC...CGTATAGAATG...TCGGTAGTGGC
cDNA3       TGGATAAGGTC...CGTATAGAATG...TCGGTAGTGGC
cDNA4       TGGATAAGGTC...CGTATAGAATG...TCGGTAGTGGC
cDNA5       TGGATAAGGTC...CGTATAGAATG...TCGGTAGTGGC
cDNA6       TGGATAAGGTC...CGTATAGAATG...TCGGTAGTGGC
cDNA7       TGGATAAGGTC...CGTATAGAATG...TCGGTAGTGGC
cDNA8       TGGATAAGGTC...CGTATAGAATG...TCGGTAGTGGC
cDNA9       TGGATGAGGTC...CGTATAGAATG...TCGGTAGTGGC
cDNA10      TGGATGAGGTC...CGTATAGAATG...TCGGTAGTGGC
cDNA11      TGGATAAGGTC...CGTATGGAATG...TCGGTAGTGGC
cDNA12      TGGATAAGGTC...CGTATGGAATG...TCGGTAGTGGC
cDNA13      TGGATAAGGTC...CGTATGGAATG...TCGGTAGTGGC
```

## E. *PCON_11363*

```
                 2235          2377          2452          2521
RNA-seq           15%           45%           32%           27%
                   |             |             |             |
PCON_11363 TATTTATGAAA...CGCTTAGAAGG...AGAATAAGACA...AGGCTAGAAGC
-------------------------------------------------------------
5d LL I            |             |             |             |
cDNA1      TATTTATGAAA...CGCTTAGAAGG...AGAATAAGACA...AGGCTAGAAGC
cDNA2      TATTTATGAAA...CGCTTAGAAGG...AGAATAAGACA...AGGCTAGAAGC
cDNA3      TATTTATGAAA...CGCTTAGAAGG...AGAATAAGACA...AGGCTAGAAGC
cDNA4      TATTTATGAAA...CGCTTAGAAGG...AGAATAAGACA...AGGCTAGAAGC
cDNA5      TATTTATGAAA...CGCTTGGAAGG...AGAATAAGACA...AGGCTAGAAGC
cDNA6      TATTTATGAAA...CGCTTGGAAGG...AGAATAAGACA...AGGCTAGAAGC
cDNA7      TATTTATGAAA...CGCTTGGAAGG...AGAATAAGACA...AGGCTGGAAGC
cDNA8      TATTTATGAAA...CGCTTGGAAGG...AGAATAAGACA...AGGCTGGAAGC
cDNA9      TATTTATGAAA...CGCTTGGAAGG...AGAATGAGACA...AGGCTGGAAGC
cDNA10     TATTTATGAAA...CGCTTGGAAGG...AGAATGAGACA...AGGCTGGAAGC
-------------------------------------------------------------
5d LL II           |             |             |             |
cDNA1      TATTTATGAAA...CGCTTAGAAGG...AGAATAAGACA...AGGCTAGAAGC
cDNA2      TATTTATGAAA...CGCTTAGAAGG...AGAATAAGACA...AGGCTAGAAGC
cDNA3      TATTTATGAAA...CGCTTAGAAGG...AGAATAAGACA...AGGCTAGAAGC
cDNA4      TATTTATGAAA...CGCTTAGAAGG...AGAATAAGACA...AGGCTAGAAGC
cDNA5      TATTTATGAAA...CGCTTAGAAGG...AGAATAAGACA...AGGCTAGAAGC
cDNA6      TATTTATGAAA...CGCTTGGAAGG...AGAATAAGACA...AGGCTAGAAGC
cDNA7      TATTTATGAAA...CGCTTGGAAGG...AGAATAAGACA...AGGCTAGAAGC
cDNA8      TATTTATGAAA...CGCTTGGAAGG...AGAATAAGACA...AGGCTAGAAGC
cDNA9      TATTTATGAAA...CGCTTGGAAGG...AGAATAAGACA...AGGCTAGAAGC
cDNA10     TATTTATGAAA...CGCTTGGAAGG...AGAATAAGACA...AGGCTGGAAGC
cDNA11     TATTTATGAAA...CGCTTGGAAGG...AGAATGAGACA...AGGCTGGAAGC
cDNA12     TATTTATGAAA...CGCTTGGAAGG...AGAATGAGACA...AGGCTGGAAGC
-------------------------------------------------------------
3d LL II           |             |             |             |
cDNA1      TATTTATGAAA...CGCTTAGAAGG...AGAATAAGACA...AGGCTAGAAGC
cDNA2      TATTTATGAAA...CGCTTAGAAGG...AGAATAAGACA...AGGCTAGAAGC
cDNA3      TATTTATGAAA...CGCTTAGAAGG...AGAATAAGACA...AGGCTAGAAGC
cDNA4      TATTTATGAAA...CGCTTAGAAGG...AGAATAAGACA...AGGCTAGAAGC
cDNA5      TATTTATGAAA...CGCTTAGAAGG...AGAATAAGACA...AGGCTAGAAGC
cDNA6      TATTTATGAAA...CGCTTAGAAGG...AGAATAAGACA...AGGCTAGAAGC
cDNA7      TATTTATGAAA...CGCTTAGAAGG...AGAATAAGACA...AGGCTAGAAGC
cDNA8      TATTTATGAAA...CGCTTAGAAGG...AGAATAAGACA...AGGCTAGAAGC
cDNA9      TATTTATGAAA...CGCTTAGAAGG...AGAATAAGACA...AGGCTAGAAGC
cDNA10     TATTTATGAAA...CGCTTAGAAGG...AGAATAAGACA...AGGCTAGAAGC
cDNA11     TATTTATGAAA...CGCTTAGAAGG...AGAATAAGACA...AGGCTAGAAGC
cDNA12     TATTTATGAAA...CGCTTGGAAGG...AGAATAAGACA...AGGCTAGAAGC
```

**Figure S2.** Verification of RNA editing sites in *P. confluens* by Sanger sequencing of DNA and cDNA clones.

The mRNA sequence predicted from genomic sequence is given on top, sequences of the cDNA clones below. The genomic sequence was verified at each site by Sanger sequencing of a cloned PCR fragment derived from genomic DNA. Three different sets of cDNA clones were used for sequencing: (1) 5d LL I: from growth in surface culture for 5d in LL, sexual development has set in. This sample was used as part of the sex I sample for RNA-seq in Traeger et al. (2013), together with RNAs from 3d and 4d. (2) 5d LL II: from growth in surface culture for 5d in LL, sexual development has set in. This sample was not used previously for RNA-seq. (3) 3d LL II: from growth in surface culture for 3d in LL, sexual development has just started. This sample was not used previously for RNA-seq.

At putative editing sites, sequences that are the same as the genomic sequence are given in blue and shaded light grey, observed A to G editing events in cDNA clones are given in red and shaded in dark grey. Positions of editing sites in gene sequence are given above each site, and percent editing in the RNA-seq data given below the position. Here, only editing sites and surrounding five nucleotides in each direction are shown. Only editing sites covered by the analyzed PCR fragments are shown, there may be more editing sites in a gene, but the others were not analyzed. For each gene fragment, 8-14 cDNA clones were sequenced per condition.

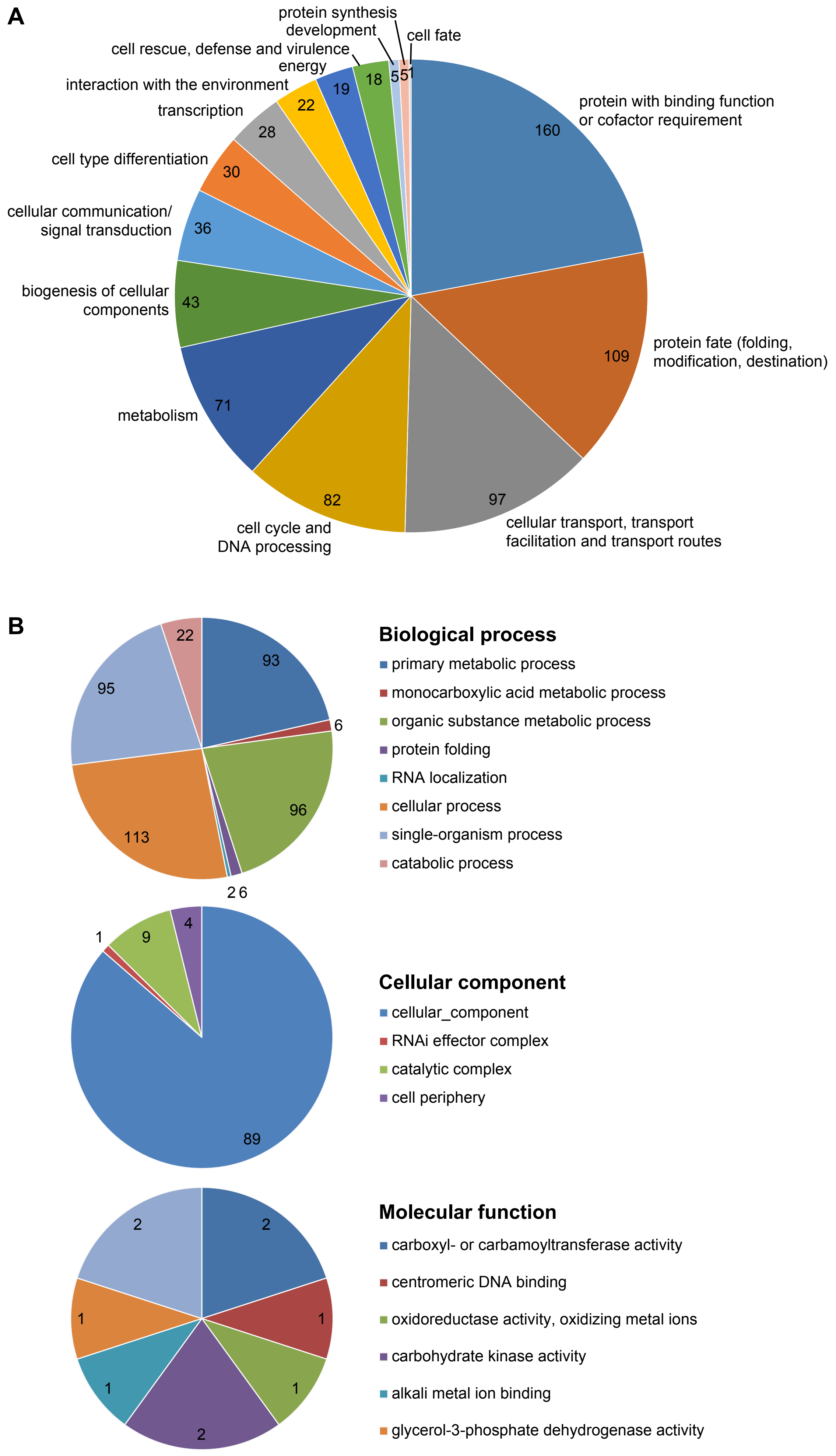


**Figure S3.** Functional classification of genes with putative editing sites in *S. macrospora*. Genes that show A-to-G changes in wild type protoperithecia only were used for the analysis. Functional classification of genes with putative editing sites was done using FungiFun 2 (Priebe et al. 2015) with the FunCat ontology (Ruepp et al. 2004) (**A**), or Ontologizer (Bauer et al. 2008) based on gene ontology (GO) annotations from UniProt (Ashburner et al. 2000, Huntley et al. 2015) (**B**). FunCat categories shown in (A) are all that are represented among the edited genes, whereas GO categories in (B) are only those that are overrepresented (adjusted p-value <0.05).

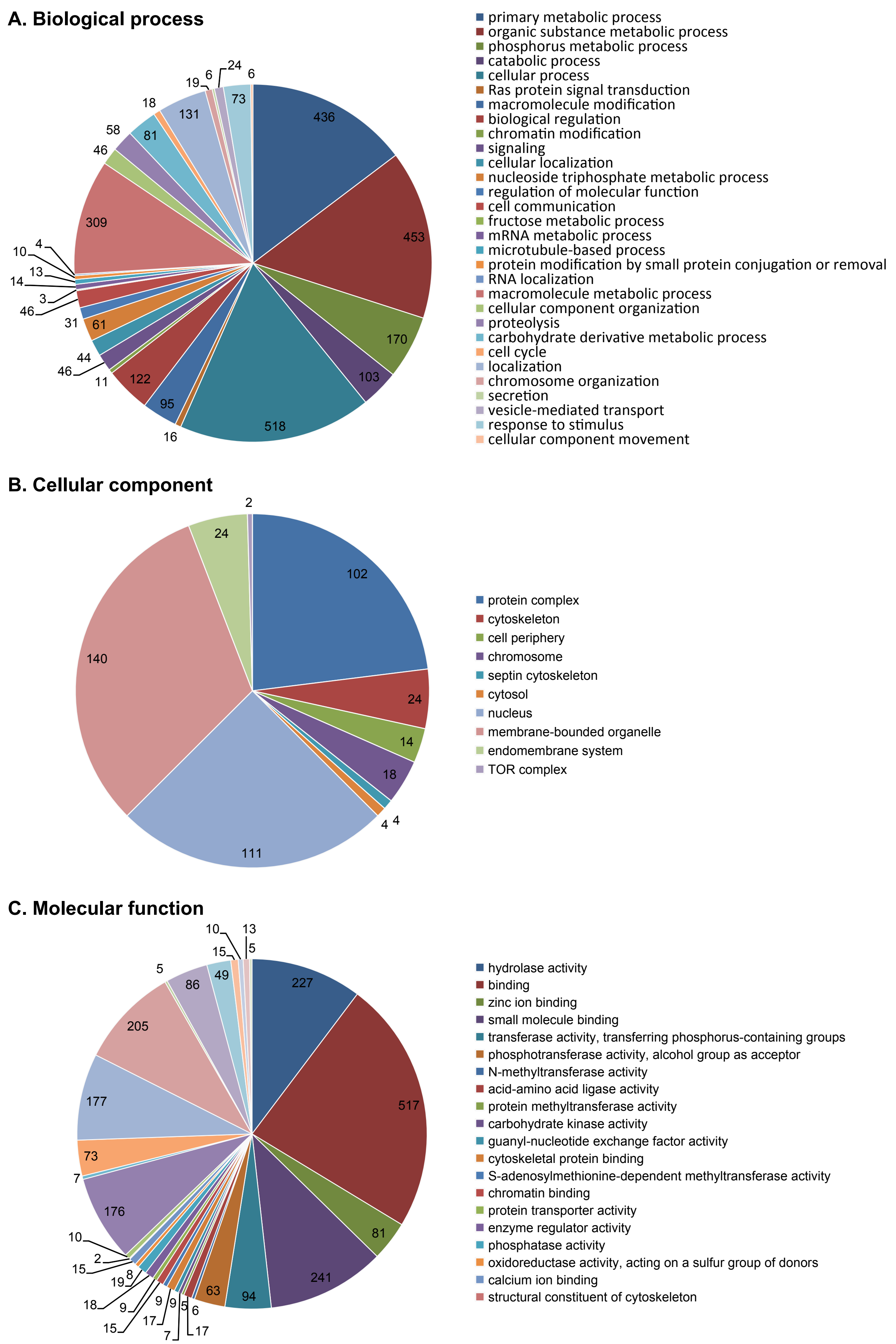


**Figure S4.** Functional classification of genes with putative editing sites in *P. confluens*. Genes that show A-to-G changes in sexual mycelium only were used for the analysis Functional classification of genes with putative editing sites was done using Ontologizer (Bauer et al. 2008) based on gene ontology (GO) annotations from UniProt (Ashburner et al. 2000, Huntley et al. 2015). Only GO categories that are overrepresented (adjusted p-value <0.05) are shown.

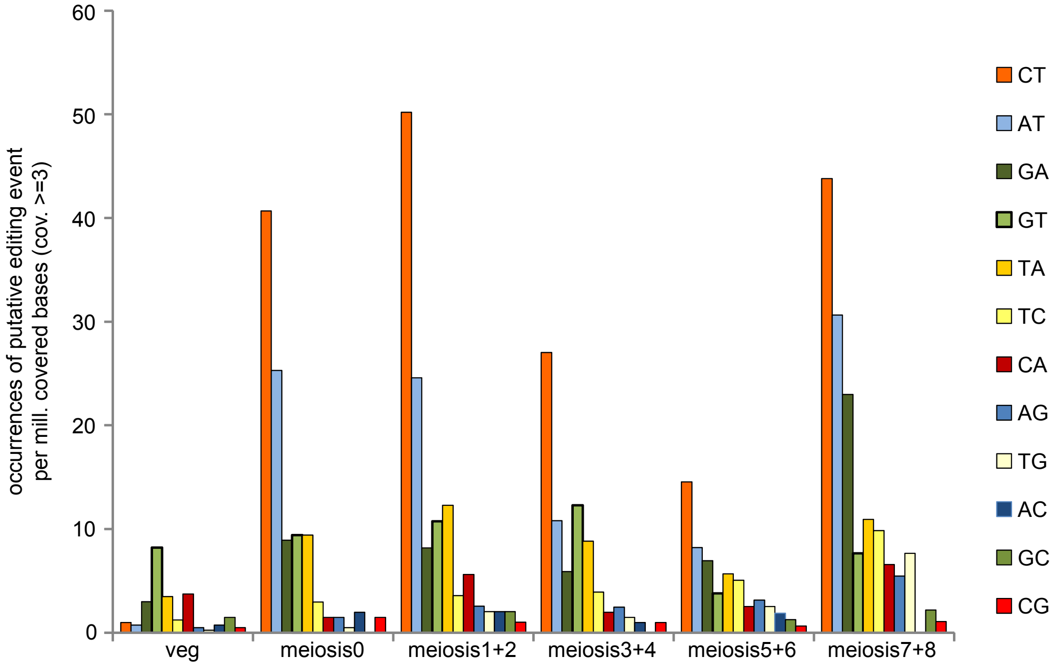


**Figure S5.** Analysis of putative editing events in *S. pombe*. RNA-seq data from three conditions were analyzed (data from Wilhelm et al. 2008, Nature 453: 1239-1243). The occurrence of base changes in annotated genes compared to genomic DNA is given as putative editing events per million covered bases, the coverage threshold was set to >=3.
