# Supplementary material for: RNA Editing During Sexual Development Occurs in Distantly Related Filamentous Ascomycetes
Source: Genome Biol Evol. 2017 Apr 1;9(4):855–68. doi: 10.1093/gbe/evx052 (PMC5381528; doi:10.1093/gbe/evx052)
Supplement: Supplementary Data [file evx052_Supp.zip › evx052_Supp/Table_S1.pdf]

Supplementary Table S1

Table S1. Oligonucleotides used in this study.

name	sequence 5'-3'	species, gene
PCON_04388_for	ACCTCTCGCAGACATGGCAACTCG	*P. confluens, PCON_04388*
PCON_04388_rev	AGAGATTTCCTGTGCTAGCTGTGC	
PCON_04707_for	TTGGAGATTCTCCTCGTGGAAAGC	*P. confluens, PCON_04707*
PCON_04707_rev	CTCCGACCCGTCCAACCATAACC	
PCON_05396_for	TTGGAGCGTGCCTTGGATCCTGG	*P. confluens, PCON_05396*
PCON_05396_rev	GAAATACCTCGACACTGTAGAAGC	
PCON_06637_for	CCGCAAACTTTTCGGCCCACTCC	*P. confluens, PCON_06637*
PCON_06637_rev	GGTTGGTCTTCTGGACAGGTTCC	
PCON_11363_for	CGGCAAATAACCGAGTTTGGAACC	*P. confluens, PCON_11363*
PCON_11363_rev	CGCTGGCACAACTTACACATGAGC	
1921-01	AGCCCATCAGCCAGGACTAC	*S. macrospora, SMAC_01921*
1921-02	CCTGACGTTGCTGCTTTGTG	
2074-04	CCTCTCAATCCGTCTCCTCC	*S. macrospora, SMAC_02074*
2074-05	CTACCCAGCAAATCGTGCCG	
2537-09	TCTTTGGCTGGCGAGGTTAG	*S. macrospora, SMAC_02573*
2537-10	GCGGCTTGGAATGTGCATAC	
4643-01	TCTACCTTGCTGGCGGGTTC	*S. macrospora, SMAC_04643*
4643-02	CTGCTACTGCTCCCATTGTG	
5974-01	GTGAACGCAAATCCGACAAG	*S. macrospora, SMAC_05974*
5974-02	CACAGCCCATATCAACAGAG	
6197-01	GGCCAAGGAGAACATCAGAC	*S. macrospora, SMAC_06197*
6197-02	CCACGCTTTGCTCCCATATC	
