# Supplementary material for: RNA Editing During Sexual Development Occurs in Distantly Related Filamentous Ascomycetes
Source: Genome Biol Evol. 2017 Apr 1;9(4):855–68. doi: 10.1093/gbe/evx052 (PMC5381528; doi:10.1093/gbe/evx052)
Supplement: Supplementary Data [file evx052_Supp.zip › evx052_Supp/Table_S7.pdf]

Supplementary Table S7

Table S7. ADAT (adenosine deaminase acting on tRNA) homologs in the genomes of *F. graminearum*, *S. macrospora*, and *P. confluens*. Data for *F. graminearum* are from Liu et al. 2016.

	ADAT1	ADAT2	ADAT3
F. graminearum	FGRRES_16992	FGRRES_11590	FGRRES_01444
S. macrospora	SMAC_02878	SMAC_06827	SMAC_02420
P. confluens	PCON_11303	PCON_14252	PCON_10066
